# Methyl 2-[2-(*tert*-but­oxy­carbonyl­amino)-1,3-benzothia­zole-6-carboxamido]­acetate

**DOI:** 10.1107/S1600536811052421

**Published:** 2011-12-14

**Authors:** Dan Gao, Xing Fang, Hai-Yang Yu, Jun-Dong Wang

**Affiliations:** a Department of Chemistry, University of Fuzhou, Fuzhou 350108, People’s Republic of China

## Abstract

In the title compound, C_16_H_19_N_3_O_5_S, the dihedral angle between the benzene ring and the carbonyl­amino group is 18.18 (2)°. In the crystal, mol­ecules form centrosymmetric dimers *via* pairs of N—H⋯N hydrogen bonds. The dimers are connected *via* N—H⋯O hydrogen bonds into a three-dimensional network..

## Related literature

For benzothia­zole derivatives with anti-tumor activity, see: Brantley *et al.* (2004 ▶); Ćaleta *et al.* (2009 ▶); Mortimer *et al.* (2006 ▶) and for benzothia­zolines with anti-tuberculous properties, see: Palmer *et al.* (1971 ▶). For related benzothia­zole structures, see: Lynch *et al.* (2002 ▶); Matković-Čalogović *et al.* (2003 ▶); Lei *et al.* (2010 ▶).
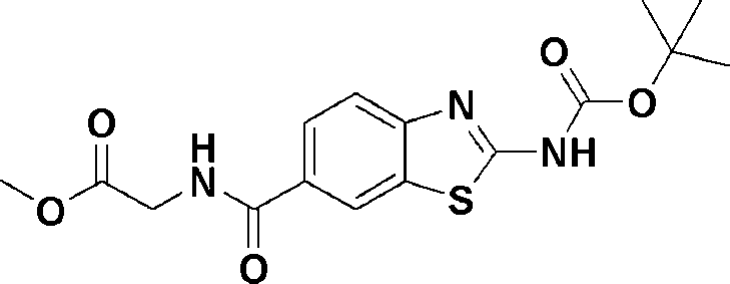


## Experimental

### 

#### Crystal data

C_16_H_19_N_3_O_5_S
*M* *_r_* = 365.41
Monoclinic, 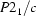

*a* = 16.861 (3) Å
*b* = 11.317 (2) Å
*c* = 9.6484 (19) Å
β = 98.94 (3)°
*V* = 1818.7 (6) Å^3^
*Z* = 4
Mo *K*α radiation
μ = 0.21 mm^−1^
*T* = 293 K
0.54 × 0.33 × 0.12 mm

#### Data collection

Rigaku Saturn 724 CCD area-detector diffractometer
Absorption correction: numerical (*NUMABS*; Higashi, 2000 ▶) *T*_min_ = 0.921, *T*_max_ = 0.975
14817 measured reflections
4176 independent reflections
3718 reflections with *I* > 2σ(*I*)
*R*_int_ = 0.054

#### Refinement

*R*[*F* ^2^ > 2σ(*F* ^2^)] = 0.083
*wR*(*F* ^2^) = 0.180
*S* = 1.26
4176 reflections
230 parameters
H-atom parameters constrained
Δρ_max_ = 0.34 e Å^−3^
Δρ_min_ = −0.23 e Å^−3^

### 

Data collection: *CrystalClear* (Rigaku, 2007 ▶); cell refinement: *CrystalClear*; data reduction: *CrystalClear*; program(s) used to solve structure: *SHELXS97* (Sheldrick, 2008 ▶); program(s) used to refine structure: *SHELXL97* (Sheldrick, 2008 ▶); molecular graphics: *ORTEX* (McArdle, 1995 ▶); software used to prepare material for publication: *SHELXL97*.

## Supplementary Material

Crystal structure: contains datablock(s) global, I. DOI: 10.1107/S1600536811052421/ff2042sup1.cif

Structure factors: contains datablock(s) I. DOI: 10.1107/S1600536811052421/ff2042Isup2.hkl

Supplementary material file. DOI: 10.1107/S1600536811052421/ff2042Isup3.cml

Additional supplementary materials:  crystallographic information; 3D view; checkCIF report

## Figures and Tables

**Table 1:** Hydrogen-bond geometry (Å, °)

*D*—H⋯*A*	*D*—H	H⋯*A*	*D*⋯*A*	*D*—H⋯*A*
N1—H1⋯N2^i^	0.86	2.16	3.005 (3)	167
N3—H3⋯O3^ii^	0.86	2.11	2.802 (3)	137

Symmetry codes: (i) 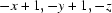; (ii) 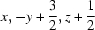.

**Fig. 1.**
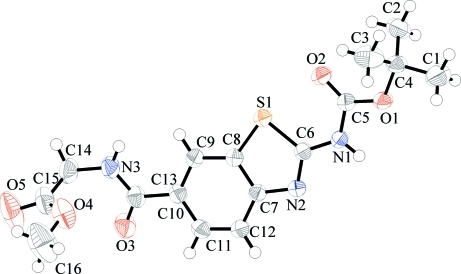
A view of the molecular structure of (I), with displacement ellipsoids drawn at the 50% probability level for non-H atoms.

## supplementary crystallographic information

### Comment

Benzothiazole is a heterocyclic compound, which contains a benzene ring and a thiazole ring, and is a component of a lot of natural products, biological pesticides, drugs, spices and so on. The benzothiazole derivatives have broad biological activities that make them play an important role in drug research and development. For example, they showed anti-tumor (Brantley *et al.*, 2004; Mortimer *et al.*, 2006;Ćaleta *et al.*, 2009) and anti-microbial activities (Palmer *et al.*, 1971). During our development of 2-aminobenzothiazole-based Urokinase-Type Plasminogen Activator (uPA) inhibitors, we synthesized the title compound (I) as an intermediate. The compound (I) has certain biological activity, and its IC50 is 780 µ*M* as uPA inhibitor itself.

The molecule structure of the title compound (I) is shown in Fig. 1. The molecular skeleton is slightly distorted from a planar conformation with the angle between benzene and thiazole rings of 1.46 (1)°. And for the substituents, the dihedral angles between the thiazole ring and *tert*-butyl carbamate is 9.15 (6)°, the dihedral angles between benzene ring and carbonylamino group is 18.18 (2)°, and the dihedral angles between carbonylamino group and methyl acetate is 79.24 (3)°.

In the crystal, there are intermolecular hydrogen bonds of N1—H1···N2 and N3—H3···O3. Where, two molecules form a pair with inversion symmetry *via* N—H···N hydrogen bonds, and the pairs form a three dimensional network *via* N—H···O hydrogen bonds. No π—π interactions are found in this structure.

### Experimental

In a 250 ml round bottom flask, the pale yellow solid of ethyl 2-(*tert*-butoxycarbonylamino) benzothiazole-6-carboxylate, N-Boc ester (3.22 g, 10 mmol) in a mixed solution of EtOH (100 ml) and 2 N aq NaOH (80 ml) were refluxed for 5 h. Then the solution was cooled with an ice bath and acidified with 1 N aq HCl, when pH<2, white floc generated and put it aside for 2 h. Then the mixture was filtered and the filter mass was washed to neutral by water and dried to afford white solid of 2-(*tert*-butoxycarbonylamino)benzothiazole-6-carboxylic acid, N-Boc acid (2.59 g, yield: 88%).

In a 100 ml round bottom flask, the mixture of N-Boc acid (705 mg, 2.4 mmol), 2-(1*H*-Benzotriazole-1-yl)-1,1,3,3-tetramethyluronium hexafluorophosphate (HBTU, 759 mg, 2 mmol), N, *N*-Diisopropylethylamine (DIEA, 310 mg, 2.4 mmol), and glycine methyl ester hydrochloride (251 mg, 2 mmol) in 20 ml dry DMF were stirred at room temperature for 20 h. Then the reaction solution was pured into 200 mL of 10% Na_2_CO_3_ solution and stirred for 1 h. The precipitate was filtered, washed with water, and dried to give the white solid of title compound (I) (591 mg, yield: 81%).

The solid was dissolved by DMF and filtered. The DMF was evaporated slowly at room temperature for 15–20 days, and colorless sheetlike crystals suitable for X-ray structure analysis were separated from the solution.

### Refinement

All hydrogen atoms were positioned geometrically and refined in a riding model approximation with *U*_iso_ (H) = 1.2 or 1.5 *U*_eq_ (C).

### Crystal data

**Table:**

C_16_H_19_N_3_O_5_S	*F*(000) = 768.0
*M**_r_* = 365.41	*D*_x_ = 1.335 Mg m^−^^3^
Monoclinic, *P*2_1_/*c*	Mo *K*α radiation, λ = 0.71073 Å
Hall symbol: -P 2ybc	Cell parameters from 5508 reflections
*a* = 16.861 (3) Å	θ = 3.0–27.5°
*b* = 11.317 (2) Å	µ = 0.21 mm^−^^1^
*c* = 9.6484 (19) Å	*T* = 293 K
β = 98.94 (3)°	Prism, colourless
*V* = 1818.7 (6) Å^3^	0.54 × 0.33 × 0.12 mm
*Z* = 4	

### Data collection

**Table:**

Rigaku Saturn 724 CCD area-detector diffractometer	4176 independent reflections
Radiation source: fine-focus sealed tube	3718 reflections with *I* > 2σ(*I*)
graphite	*R*_int_ = 0.054
Detector resolution: 28.5714 pixels mm^-1^	θ_max_ = 27.5°, θ_min_ = 3.0°
dtprofit.ref scans	*h* = −20→21
Absorption correction: numerical (*NUMABS*; Higashi, 2000)	*k* = −14→14
*T*_min_ = 0.921, *T*_max_ = 0.975	*l* = −12→12
14817 measured reflections	

### Refinement

**Table:**

Refinement on *F*^2^	Primary atom site location: structure-invariant direct methods
Least-squares matrix: full	Secondary atom site location: difference Fourier map
*R*[*F*^2^ > 2σ(*F*^2^)] = 0.083	Hydrogen site location: inferred from neighbouring sites
*wR*(*F*^2^) = 0.180	H-atom parameters constrained
*S* = 1.26	*w* = 1/[σ^2^(*F*_o_^2^) + (0.0486*P*)^2^ + 1.240*P*] where *P* = (*F*_o_^2^ + 2*F*_c_^2^)/3
4176 reflections	(Δ/σ)_max_ < 0.001
230 parameters	Δρ_max_ = 0.34 e Å^−^^3^
0 restraints	Δρ_min_ = −0.23 e Å^−^^3^

### Special details

**Table:**

Geometry. All e.s.d.'s (except the e.s.d. in the dihedral angle between two l.s. planes) are estimated using the full covariance matrix. The cell e.s.d.'s are taken into account individually in the estimation of e.s.d.'s in distances, angles and torsion angles; correlations between e.s.d.'s in cell parameters are only used when they are defined by crystal symmetry. An approximate (isotropic) treatment of cell e.s.d.'s is used for estimating e.s.d.'s involving l.s. planes.
Refinement. Refinement of *F*^2^ against ALL reflections. The weighted *R*-factor *wR* and goodness of fit *S* are based on *F*^2^, conventional *R*-factors *R* are based on *F*, with *F* set to zero for negative *F*^2^. The threshold expression of *F*^2^ > σ(*F*^2^) is used only for calculating *R*-factors(gt) *etc*. and is not relevant to the choice of reflections for refinement. *R*-factors based on *F*^2^ are statistically about twice as large as those based on *F*, and *R*- factors based on ALL data will be even larger.

### Fractional atomic coordinates and isotropic or equivalent isotropic displacement parameters (Å^2^)

**Table:**

	*x*	*y*	*z*	*U*_iso_*/*U*_eq_	
S1	0.36614 (4)	0.72248 (7)	0.16296 (8)	0.0467 (2)	
O3	0.05637 (13)	0.7065 (2)	−0.3160 (2)	0.0637 (7)	
O4	−0.06380 (16)	0.6314 (3)	−0.0995 (3)	0.0789 (8)	
O2	0.49650 (13)	0.7178 (2)	0.3633 (2)	0.0574 (6)	
O1	0.60458 (12)	0.59926 (19)	0.3591 (2)	0.0479 (5)	
O5	−0.16027 (18)	0.7431 (4)	−0.2127 (5)	0.1271 (15)	
N1	0.50500 (13)	0.5954 (2)	0.1795 (2)	0.0415 (6)	
H1	0.5363	0.5490	0.1426	0.050*	
N2	0.40231 (13)	0.5568 (2)	−0.0059 (2)	0.0377 (5)	
N3	0.05095 (15)	0.7905 (3)	−0.1077 (3)	0.0524 (7)	
H3	0.0756	0.8095	−0.0259	0.063*	
C9	0.21789 (16)	0.7285 (3)	−0.0217 (3)	0.0431 (7)	
H9	0.1985	0.7890	0.0290	0.052*	
C3	0.5917 (3)	0.5968 (4)	0.6084 (4)	0.0761 (12)	
H3A	0.5441	0.6448	0.5945	0.114*	
H3B	0.6199	0.6089	0.7017	0.114*	
H3C	0.5770	0.5151	0.5962	0.114*	
C15	−0.0922 (2)	0.7299 (4)	−0.1594 (4)	0.0696 (11)	
C16	−0.1190 (3)	0.5336 (5)	−0.1009 (7)	0.133 (2)	
H16A	−0.1640	0.5574	−0.0578	0.199*	
H16B	−0.0922	0.4684	−0.0498	0.199*	
H16C	−0.1373	0.5100	−0.1960	0.199*	
C1	0.7200 (2)	0.5557 (4)	0.5209 (4)	0.0855 (14)	
H1A	0.7053	0.4740	0.5087	0.128*	
H1B	0.7497	0.5673	0.6132	0.128*	
H1C	0.7526	0.5781	0.4521	0.128*	
C6	0.43024 (16)	0.6160 (2)	0.1075 (3)	0.0372 (6)	
C7	0.32479 (16)	0.5921 (2)	−0.0557 (3)	0.0363 (6)	
C10	0.17080 (16)	0.6823 (3)	−0.1401 (3)	0.0409 (6)	
C8	0.29461 (16)	0.6830 (2)	0.0201 (3)	0.0384 (6)	
C12	0.27640 (17)	0.5443 (3)	−0.1727 (3)	0.0427 (7)	
H12	0.2951	0.4828	−0.2228	0.051*	
C11	0.20071 (17)	0.5895 (3)	−0.2130 (3)	0.0440 (7)	
H11	0.1684	0.5575	−0.2909	0.053*	
C13	0.08877 (18)	0.7271 (3)	−0.1946 (3)	0.0460 (7)	
C5	0.53326 (17)	0.6445 (3)	0.3083 (3)	0.0419 (6)	
C4	0.64493 (19)	0.6309 (3)	0.5033 (3)	0.0484 (7)	
C14	−0.03095 (19)	0.8269 (4)	−0.1511 (4)	0.0637 (10)	
H14A	−0.0350	0.8638	−0.2427	0.076*	
H14B	−0.0441	0.8864	−0.0860	0.076*	
C2	0.6663 (2)	0.7601 (3)	0.5084 (4)	0.0668 (10)	
H2A	0.6974	0.7777	0.4356	0.100*	
H2B	0.6972	0.7785	0.5980	0.100*	
H2C	0.6181	0.8065	0.4947	0.100*	

### Atomic displacement parameters (Å^2^)

**Table:**

	*U*^11^	*U*^22^	*U*^33^	*U*^12^	*U*^13^	*U*^23^
S1	0.0417 (4)	0.0507 (5)	0.0453 (4)	0.0087 (3)	−0.0005 (3)	−0.0150 (3)
O3	0.0521 (13)	0.096 (2)	0.0399 (12)	0.0100 (13)	−0.0022 (10)	−0.0002 (12)
O4	0.0595 (16)	0.095 (2)	0.0791 (18)	−0.0064 (15)	0.0023 (14)	0.0225 (16)
O2	0.0514 (13)	0.0652 (15)	0.0529 (13)	0.0140 (11)	−0.0002 (10)	−0.0218 (11)
O1	0.0480 (11)	0.0523 (13)	0.0397 (11)	0.0121 (9)	−0.0045 (9)	−0.0131 (9)
O5	0.0533 (18)	0.141 (3)	0.172 (4)	0.0003 (19)	−0.029 (2)	0.035 (3)
N1	0.0374 (12)	0.0463 (14)	0.0399 (12)	0.0078 (10)	0.0028 (10)	−0.0081 (10)
N2	0.0387 (12)	0.0367 (12)	0.0376 (12)	0.0003 (9)	0.0053 (10)	−0.0051 (10)
N3	0.0409 (14)	0.0684 (19)	0.0456 (14)	0.0096 (12)	−0.0003 (11)	−0.0011 (13)
C9	0.0388 (15)	0.0472 (17)	0.0439 (15)	0.0045 (12)	0.0080 (12)	−0.0043 (13)
C3	0.103 (3)	0.079 (3)	0.0425 (19)	−0.007 (2)	0.003 (2)	0.0012 (19)
C15	0.0423 (19)	0.099 (3)	0.065 (2)	0.0063 (19)	0.0001 (16)	0.007 (2)
C16	0.097 (4)	0.135 (5)	0.160 (6)	−0.038 (4)	0.000 (4)	0.054 (5)
C1	0.085 (3)	0.092 (3)	0.067 (2)	0.040 (2)	−0.029 (2)	−0.028 (2)
C6	0.0377 (14)	0.0371 (14)	0.0367 (14)	0.0016 (11)	0.0049 (11)	−0.0013 (11)
C7	0.0362 (14)	0.0352 (14)	0.0377 (14)	−0.0004 (11)	0.0064 (11)	−0.0003 (11)
C10	0.0381 (14)	0.0473 (17)	0.0374 (14)	−0.0010 (12)	0.0064 (11)	0.0013 (12)
C8	0.0372 (14)	0.0384 (15)	0.0392 (14)	0.0007 (11)	0.0050 (11)	−0.0016 (12)
C12	0.0445 (16)	0.0406 (16)	0.0428 (15)	0.0016 (12)	0.0059 (12)	−0.0043 (13)
C11	0.0441 (16)	0.0492 (17)	0.0371 (14)	−0.0039 (13)	0.0013 (12)	−0.0023 (13)
C13	0.0409 (16)	0.0563 (19)	0.0400 (15)	0.0027 (13)	0.0037 (12)	0.0069 (14)
C5	0.0397 (15)	0.0447 (16)	0.0407 (15)	0.0016 (12)	0.0048 (12)	−0.0061 (13)
C4	0.0539 (18)	0.0501 (18)	0.0372 (15)	0.0083 (14)	−0.0057 (13)	−0.0104 (13)
C14	0.0473 (18)	0.074 (3)	0.067 (2)	0.0189 (17)	0.0000 (16)	0.0019 (19)
C2	0.065 (2)	0.059 (2)	0.071 (2)	−0.0095 (17)	−0.0060 (18)	−0.0116 (19)

### Geometric parameters (Å, °)

**Table:**

S1—C8	1.742 (3)	C3—H3C	0.9600
S1—C6	1.757 (3)	C15—C14	1.501 (6)
O3—C13	1.235 (4)	C16—H16A	0.9600
O4—C15	1.312 (5)	C16—H16B	0.9600
O4—C16	1.444 (5)	C16—H16C	0.9600
O2—C5	1.207 (3)	C1—C4	1.513 (4)
O1—C5	1.329 (3)	C1—H1A	0.9600
O1—C4	1.494 (3)	C1—H1B	0.9600
O5—C15	1.191 (4)	C1—H1C	0.9600
N1—C6	1.362 (3)	C7—C12	1.395 (4)
N1—C5	1.377 (3)	C7—C8	1.403 (4)
N1—H1	0.8600	C10—C11	1.401 (4)
N2—C6	1.307 (3)	C10—C13	1.490 (4)
N2—C7	1.380 (3)	C12—C11	1.373 (4)
N3—C13	1.338 (4)	C12—H12	0.9300
N3—C14	1.440 (4)	C11—H11	0.9300
N3—H3	0.8600	C4—C2	1.505 (5)
C9—C10	1.389 (4)	C14—H14A	0.9700
C9—C8	1.393 (4)	C14—H14B	0.9700
C9—H9	0.9300	C2—H2A	0.9600
C3—C4	1.506 (5)	C2—H2B	0.9600
C3—H3A	0.9600	C2—H2C	0.9600
C3—H3B	0.9600		
			
C8—S1—C6	88.06 (13)	N2—C7—C8	115.6 (2)
C15—O4—C16	117.2 (3)	C12—C7—C8	119.5 (2)
C5—O1—C4	120.4 (2)	C9—C10—C11	119.4 (3)
C6—N1—C5	123.7 (2)	C9—C10—C13	122.9 (3)
C6—N1—H1	118.2	C11—C10—C13	117.7 (3)
C5—N1—H1	118.2	C9—C8—C7	121.1 (3)
C6—N2—C7	110.0 (2)	C9—C8—S1	129.2 (2)
C13—N3—C14	120.0 (3)	C7—C8—S1	109.7 (2)
C13—N3—H3	120.0	C11—C12—C7	119.0 (3)
C14—N3—H3	120.0	C11—C12—H12	120.5
C10—C9—C8	119.1 (3)	C7—C12—H12	120.5
C10—C9—H9	120.5	C12—C11—C10	121.9 (3)
C8—C9—H9	120.5	C12—C11—H11	119.1
C4—C3—H3A	109.5	C10—C11—H11	119.1
C4—C3—H3B	109.5	O3—C13—N3	120.8 (3)
H3A—C3—H3B	109.5	O3—C13—C10	121.3 (3)
C4—C3—H3C	109.5	N3—C13—C10	117.9 (3)
H3A—C3—H3C	109.5	O2—C5—O1	126.9 (3)
H3B—C3—H3C	109.5	O2—C5—N1	123.0 (3)
O5—C15—O4	123.9 (4)	O1—C5—N1	110.1 (2)
O5—C15—C14	122.7 (4)	O1—C4—C2	109.6 (3)
O4—C15—C14	113.4 (3)	O1—C4—C3	109.4 (3)
O4—C16—H16A	109.5	C2—C4—C3	113.1 (3)
O4—C16—H16B	109.5	O1—C4—C1	102.8 (2)
H16A—C16—H16B	109.5	C2—C4—C1	110.5 (3)
O4—C16—H16C	109.5	C3—C4—C1	110.9 (3)
H16A—C16—H16C	109.5	N3—C14—C15	115.3 (3)
H16B—C16—H16C	109.5	N3—C14—H14A	108.4
C4—C1—H1A	109.5	C15—C14—H14A	108.4
C4—C1—H1B	109.5	N3—C14—H14B	108.4
H1A—C1—H1B	109.5	C15—C14—H14B	108.4
C4—C1—H1C	109.5	H14A—C14—H14B	107.5
H1A—C1—H1C	109.5	C4—C2—H2A	109.5
H1B—C1—H1C	109.5	C4—C2—H2B	109.5
N2—C6—N1	121.6 (2)	H2A—C2—H2B	109.5
N2—C6—S1	116.6 (2)	C4—C2—H2C	109.5
N1—C6—S1	121.8 (2)	H2A—C2—H2C	109.5
N2—C7—C12	124.9 (2)	H2B—C2—H2C	109.5
			
C16—O4—C15—O5	0.7 (7)	C8—C7—C12—C11	1.5 (4)
C16—O4—C15—C14	179.2 (4)	C7—C12—C11—C10	0.1 (4)
C7—N2—C6—N1	178.2 (2)	C9—C10—C11—C12	−1.6 (5)
C7—N2—C6—S1	−1.2 (3)	C13—C10—C11—C12	178.3 (3)
C5—N1—C6—N2	−171.5 (3)	C14—N3—C13—O3	5.3 (5)
C5—N1—C6—S1	7.8 (4)	C14—N3—C13—C10	−174.5 (3)
C8—S1—C6—N2	0.2 (2)	C9—C10—C13—O3	161.8 (3)
C8—S1—C6—N1	−179.1 (3)	C11—C10—C13—O3	−18.2 (5)
C6—N2—C7—C12	−178.0 (3)	C9—C10—C13—N3	−18.4 (5)
C6—N2—C7—C8	1.8 (3)	C11—C10—C13—N3	161.7 (3)
C8—C9—C10—C11	1.4 (4)	C4—O1—C5—O2	5.0 (5)
C8—C9—C10—C13	−178.6 (3)	C4—O1—C5—N1	−174.9 (2)
C10—C9—C8—C7	0.3 (4)	C6—N1—C5—O2	−6.2 (5)
C10—C9—C8—S1	−179.6 (2)	C6—N1—C5—O1	173.7 (3)
N2—C7—C8—C9	178.4 (3)	C5—O1—C4—C2	−65.7 (4)
C12—C7—C8—C9	−1.8 (4)	C5—O1—C4—C3	58.8 (4)
N2—C7—C8—S1	−1.7 (3)	C5—O1—C4—C1	176.7 (3)
C12—C7—C8—S1	178.2 (2)	C13—N3—C14—C15	71.6 (4)
C6—S1—C8—C9	−179.3 (3)	O5—C15—C14—N3	−168.8 (4)
C6—S1—C8—C7	0.8 (2)	O4—C15—C14—N3	12.7 (5)
N2—C7—C12—C11	−178.6 (3)		

### Hydrogen-bond geometry (Å, °)

**Table:**

*D*—H···*A*	*D*—H	H···*A*	*D*···*A*	*D*—H···*A*
N1—H1···N2^i^	0.86	2.16	3.005 (3)	167.
N3—H3···O3^ii^	0.86	2.11	2.802 (3)	137.

Symmetry codes: (i) −*x*+1, −*y*+1, −*z*; (ii) *x*, −*y*+3/2, *z*+1/2.
